# A reconfigurable on-line learning spiking neuromorphic processor comprising 256 neurons and 128K synapses

**DOI:** 10.3389/fnins.2015.00141

**Published:** 2015-04-29

**Authors:** Ning Qiao, Hesham Mostafa, Federico Corradi, Marc Osswald, Fabio Stefanini, Dora Sumislawska, Giacomo Indiveri

**Affiliations:** Institute of Neuroinformatics, University of Zurich and ETH ZurichZurich, Switzerland

**Keywords:** spike-based learning, Spike-Timing Dependent Plasticity (STDP), real-time, analog VLSI, Winner-Take-All (WTA), attractor network, asynchronous, brain-inspired computing

## Abstract

Implementing compact, low-power artificial neural processing systems with real-time on-line learning abilities is still an open challenge. In this paper we present a full-custom mixed-signal VLSI device with neuromorphic learning circuits that emulate the biophysics of real spiking neurons and dynamic synapses for exploring the properties of computational neuroscience models and for building brain-inspired computing systems. The proposed architecture allows the on-chip configuration of a wide range of network connectivities, including recurrent and deep networks, with short-term and long-term plasticity. The device comprises 128 K analog synapse and 256 neuron circuits with biologically plausible dynamics and bi-stable spike-based plasticity mechanisms that endow it with on-line learning abilities. In addition to the analog circuits, the device comprises also asynchronous digital logic circuits for setting different synapse and neuron properties as well as different network configurations. This prototype device, fabricated using a 180 nm 1P6M CMOS process, occupies an area of 51.4 mm^2^, and consumes approximately 4 mW for typical experiments, for example involving attractor networks. Here we describe the details of the overall architecture and of the individual circuits and present experimental results that showcase its potential. By supporting a wide range of cortical-like computational modules comprising plasticity mechanisms, this device will enable the realization of intelligent autonomous systems with on-line learning capabilities.

## 1. Introduction

Recent advances in neural network modeling and theory, combined with advances in technology and computing power, are producing impressive results in a wide range of application domains. For example, large-scale deep-belief neural networks and convolutional networks now represent the state-of-the-art for speech recognition and image segmentation applications (Mohamed et al., 2012; Farabet et al., 2013). However, the mostly sequential and synchronous clocked nature of conventional computing platforms is not optimally suited for the implementation of these types of massively parallel neural network architectures. For this reason a new generation of custom neuro-computing hardware systems started to emerge. These systems are typically composed of custom Very Large Scale Integration (VLSI) chips that either contain digital processing cores with dedicated memory structures and communication schemes optimized for spiking neural networks architectures (Wang et al., 2013; Furber et al., 2014; Neil and Liu, 2014), or full-custom digital circuit solutions that implement large arrays of spiking neurons with programmable synaptic connections (Merolla et al., 2014). While these devices and systems have high potential for solving machine learning tasks and applied research problems, they do not emulate directly the dynamics of real neural systems.

At the other end of the spectrum, neuromorphic engineering researchers have been developing hardware implementations of detailed neural models, using mixed signal analog-digital circuits to reproduce faithfully neural and synaptic dynamics, in a basic research effort to understand the principles of neural computation in physical hardware systems (Douglas et al., 1995; Liu et al., 2002; Chicca et al., 2014). By studying the physics of computation of neural systems, and reproducing it through the physics of transistors biased in the subthreshold regime (Liu et al., 2002), neuromorphic engineering seeks to emulate biological neural computing systems efficiently, using the least amount of power and silicon real-estate possible. Examples of biophysically realistic neural electronic circuits built following this approach range from models of single neurons (Mahowald and Douglas, 1991; Farquhar and Hasler, 2005; Hynna and Boahen, 2007; van Schaik et al., 2010), to models of synaptic dynamics (Liu, 2003; Bartolozzi and Indiveri, 2007a; Xu et al., 2007), to auditory/visual sensory systems (Sarpeshkar et al., 1996; van Schaik and Meddis, 1999; Zaghloul and Boahen, 2004; Costas-Santos et al., 2007; Liu and Delbruck, 2010), to reconfigurable spiking neural network architectures with learning and plasticity (Giulioni et al., 2008; Hsieh and Tang, 2012; Ramakrishnan et al., 2012; Yu et al., 2012; Chicca et al., 2014).

In this paper we propose to combine the basic research efforts with the applied research ones, by presenting a VLSI architecture that can be used to both carry out research experiments in computational neuroscience, and to develop application solutions for practical tasks. The architecture proposed comprises electronic neuromorphic circuits that directly emulate the physics of real neurons and synapses to faithfully reproduce their adaptive and dynamic behavior, together with digital logic circuits that can set both the properties of the individual synapse and neuron elements as well as the topology of the neural network. In particular, this architecture has been developed to implement spike-based adaptation and plasticity mechanisms, and to carry out on-chip on-line learning for tasks that require the system to adapt to the changes in the environment it interacts with. Given these characteristics, including the ability to arbitrarily reconfigure the network topology also at run-time, we named this device the Reconfigurable On-line Learning Spiking Neuromorphic Processor (ROLLS neuromorphic processor).

The main novelty of the work proposed, compared to previous analogous approaches (Indiveri et al., 2006; Giulioni et al., 2008; Ramakrishnan et al., 2012; Yu et al., 2012) consists in the integration of analog bi-stable learning synapse circuits with asynchronous digital logic cells and in the embedding of these mixed-signal blocks in a large multi-neuron architecture. The combination of analog and digital circuits, with both analog and digital memory elements, within the same block provides the device with an important set of programmable features, including the ability to configure arbitrary network connectivity schemes. At the analog circuit design level, we present improvements in the neuron and spike-based learning synapses over previously proposed ones (Indiveri et al., 2011; Chicca et al., 2014), which extend their range of behaviors and significantly reduce device mismatch effects. At the system application level we demonstrate, for the first time, both computational neuroscience models of attractor networks and image classification neural networks implemented exclusively on custom mixed-signal analog-digital neuromorphic hardware, with no extra pre- or post-processing done in software. In the next section we describe the ROLLS neuromorphic processor system-level block diagram, highlighting its dynamic and spike-based learning features. In Section 2.2 we describe in detail the circuits that are present in each building block, and in Section 3 we present system level experimental results showcasing examples of both computational neuroscience models and machine vision pattern recognition tasks. Finally, in Sections 4, 5 we discuss the results obtained and summarize our contribution with concluding remarks.

## 2. Materials and methods

### 2.1. The neuromorphic processor architecture

**Figure 1:**
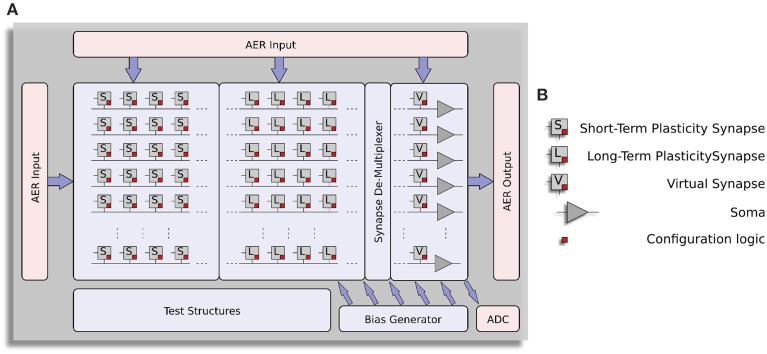
**Architecture of ROLLS neuromorphic processor. (A)** Block diagram of the architecture, showing two distinct synapse arrays (short-term plasticity and long-term plasticity synapses), an additional row of synapses (virtual synapses) and a row of neurons (somas). A synapse de-multiplexer block is used to connect the rows from the synapse arrays to the neurons (see main text for details). Peripheral circuits include asynchronous digital AER logic blocks, an Analog-to-Digital converter, and a programmable on-chip bias-generator. **(B)** Block-diagram legend.

The block-diagram of the ROLLS neuromorphic processor architecture is shown in Figure 1. The device comprises a configurable array of synapse circuits that produce biologically realistic response properties and spiking neurons that can exhibit a wide range of realistic behaviors. Specifically, this device comprises a row of 256×1 silicon neuron circuits, an array of 256×256 learning synapse circuits for modeling long-term plasticity mechanisms, an array of 256×256 programmable synapses with short-term plasticity circuits, a 256×2 row of linear integrator filters denoted as “virtual synapses” for modeling excitatory and inhibitory synapses that have shared synaptic weights and time constants, and additional peripheral analog/digital Input/Output (I/O) circuits for both receiving and transmitting spikes in real-time off-chip.

**Figure 2:**
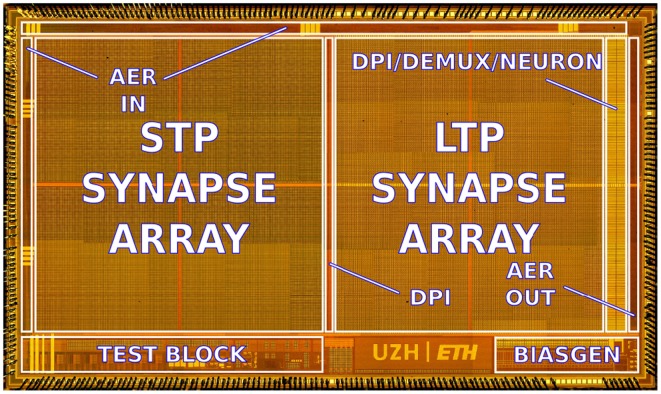
**Micro-photograph of the ROLLS neuromorphic processor**. The chip was fabricated using a 180 nm CMOS process and occupies an area of 51.4 mm^2^, comprising 12.2 million transistors.

The ROLLS neuromorphic processor was fabricated using a standard 180 nm Complementary Metal-Oxide-Semiconductor (CMOS) 1P6M process. It occupies an areas of 51.4 mm^2^ and has approximately 12.2 million transistors. The die photo of the chip is shown in Figure 2. The area distribution of main circuit blocks is shown in Table 1. The silicon neurons contain circuits that implement a model of the adaptive exponential Integrate-and-Fire (I&F) neuron (Brette and Gerstner, 2005), post-synaptic learning circuits used to implement the spike-based weight-update/plasticity mechanism in the array of long-term plasticity synapses, and analog circuits that model homeostatic synaptic scaling mechanisms operating on very long time scales (Rovere et al., 2014). The array of long-term plasticity synapses comprises pre-synaptic spike-based learning circuits with bi-stable synaptic weights, that can undergo either Long-Term Potentiation (LTP) or Long-Term Depression (LTD), (see Section 2.1.2 for details). The array of Short-Term Plasticity (STP) synapses comprises synapses with programmable weights and STP circuits that reproduce short-term adaptation dynamics. Both arrays contain analog integrator circuits that implement faithful models of synaptic temporal dynamics (see Section 2.1.1). Digital configuration logic in each of the synapse and neuron circuits allows the user to program the properties of the synapses, the topology of the network, and the properties of the neurons.

**Table 1:** **Circuits area distribution**.

**Circuit**	**Dimensions (μ*m* × μ*m*)**	**Number**	**Total area:**	**(*mm*^2^)**	**(%)**
Neuron	55.69×16.48	256		0.235	0.47
Post-synaptic learning	39.09×16.48	256		0.165	0.32
LTP synapse	15.3×16.48	64 k		16.147	31.41
STP synapse	16.24×16.48	64 k		17.129	33.32
Virtual synapse	35.6×16.48	512		0.300	0.58
Synapse de-mux	49.56×4389.4	1		0.218	0.42
AER in (columns)	8770×154	1		0.135	0.26
AER in (rows)	112×4357	1		0.488	0.95
AER out	166.2×4274.9	1		0.710	1.38
BiasGen	539.5×1973	1		1.064	2.07

*The remaining area used in the chip is occupied by the pads and additional test structures*.

The architecture comprises also a “synapse de-multiplexer” static logic circuit, which allows the user to choose how many rows of plastic synapses should be connected to the neurons. It is a programmable switch-matrix that configures the connectivity between the synapse rows and the neuron columns. By default, each of the 256 rows of 1×512 synapses is connected to its corresponding neuron. By changing the circuit control bits it is possible to allocate multiple synapse rows to the neurons, thereby disconnecting and sacrificing the unused neurons. In the extreme case all 256×512 synapses are assigned to a single neuron, and the remaining 255 neurons remain unused.

An on-chip programmable bias generator, optimized for subthreshold circuits (Delbruck et al., 2010) is used to set all of the bias currents that control the parameters of the synapses and neurons (such as time constants, leak currents, *etc*.).

An Analog to Digital Converter (ADC) circuit converts the subthreshold currents produced by selected synapse and neuron circuits into a stream of voltage pulses, using a linear pulse-frequency-modulation scheme, and transmits them off-chip as digital signals.

Finally, peripheral asynchronous I/O logic circuits are used for receiving input spikes and transmitting output ones, using the Address-Event Representation (AER) communication protocol (Deiss et al., 1998; Boahen, 2000).

#### 2.1.1. Synapse temporal dynamics

In the ROLLS neuromorphic processor all synapses process input spikes in real-time, as they arrive. Similarly the neurons transmit the spikes they produce immediately, as they are generated. In these types of architectures time represents itself and input data is processed instantaneously. There is no virtualization of time and no mechanism for storing partial results in memory banks. As a consequence, the circuits must operate with time-constants that are well-matched to those of the signals they are designed to process. Since this device is intended to be used in behaving systems that interact with the environment in natural real-world scenarios, it is important to design circuits that can implement a wide range of time constants, including very slow, biologically plausible, ones. To achieve this, and to model neural dynamics with biologically plausible time constants, we used the Differential Pair Integrator (DPI) (Bartolozzi and Indiveri, 2007b). This is a current-mode log-domain integrator. When biased in the subthreshold regime, this circuit can obtain long time constants, even with relatively small and compact capacitors. For example, in the 180 nm technology used, with a capacitor of 1 pF, we could obtain time constants of the order of tens of milliseconds without resorting to any advanced design techniques. However, to realize even longer time constants (e.g., of the order of hundreds of milliseconds), we used a shifted-source biasing technique, as described in Linares-Barranco and Serrano-Gotarredona (2003).

The synapse circuits in the two synapse arrays of the ROLLS neuromorphic processor convert input voltage spikes into output currents which have non-linear dynamics, due to their adaptation or learning features. In addition, to model the synapse temporal dynamics, the currents produced by the circuit elements in the array are further integrated by a linear temporal filter. If we assume that all the synapses in an array have the same temporal dynamics (i.e., share the same time constants), then we can exploit Kirchhoff's current law and sum the output currents of all synapses in a row into a single DPI circuit. This allows us to save a significant amount of silicon real-estate, as we can use only one DPI per row, in each array. In particular, we use one excitatory DPI in the long-term plasticity array configured to produce time constants of the order of hundreds of milliseconds, to model the dynamics of N-Methyl-D-Aspartate (NMDA) receptors, and two DPI circuits (one for excitatory and one for inhibitory synaptic dynamics) in the STP array, configured with time constants of the order of tens of milliseconds, to model the dynamics of AMPA and GABA receptors, respectively.

We use the same principle for the 256×2 “virtual synapse” integrators in the architecture. These circuits comprise two DPI integrators per row (one for the excitatory synapse and one for the inhibitory one) with fixed sets of weights and shared time-constant parameters, biased to operate in their linear operating range. By time-multiplexing input spikes to a single virtual synapse we can model the effect of multiple independent inputs to the targeted neuron. For example, by stimulating the DPI with a single 10 KHz spike train, we can model the effect of 1000 synapses receiving a 10 Hz input spike train.

#### 2.1.2. The spike-based learning algorithm

Many models of Spike-Timing Dependent Plasticity (STDP) have been proposed in the computational neuroscience literature (Abbott and Nelson, 2000; Markram et al., 2012). However, a growing body of evidence is revealing that learning algorithms based on spike-timing alone cannot account for all of the phenomenology observed neurophysiological experiments (Lisman and Spruston, 2010), have poor memory retention performance (Billings and van Rossum, 2009), and require additional mechanisms to learn both spike-time correlations and mean firing rates in the input patterns (Senn, 2002).

For this reason, we chose to implement the spike-driven synaptic plasticity rule proposed by Brader et al. (2007), which has been shown to reproduce many of the behaviors observed in biology, and has performance characteristics that make it competitive with the state-of-the-art machine learning methods (Brader et al., 2007). This algorithm does not rely on spike-timing alone. It updates the synaptic weights according to the timing of the pre-synaptic spike, the state of the post-synaptic neuron's membrane potential, and its recent spiking activity. It assumes that the synaptic weights are bounded, and that, on long time-scales, they converge to either a high state, or a low one. However, in order to avoid updating all synapses in exactly the same way, this algorithm requires a stochastic weight update mechanism (see Brader et al., 2007 for details).

The requirements and features of this algorithm make it particularly well-suited for neuromorphic hardware implementation: the bi-stability feature removes the problematic need of storing precise analog variables on long-time scales, while the probabilistic weight update requirement can be obtained by simply exploiting the variability in the input spike trains (typically produced by a Poisson process) and the variability in the post-synaptic neuron's membrane potential (typically driven by noisy sensory inputs).

The weight-update rule for a given synapse *i* is governed by the following equations, which are evaluated upon the arrival of each pre-synaptic spike:

(1) $$\left\{ \begin{array}{l} \;w_{i}=w_{i}+\Delta w^{+}\text{ if }V_{mem}(t_{pre})>\theta_{mem}\text{ and}\\ \;\theta_{1}<Ca(t_{pre})<\theta_{3}\\ w_{i}=w_{i}-\Delta w^{-}\text{ if }V_{mem}(t_{pre})<\theta_{mem}\text{ and}\\ \;\theta_{1}<Ca(t_{pre})<\theta_{2} \end{array}\right.$$

where *w_i_* represents an internal variable that encodes the bi-stale synaptic weight; the terms Δ*w*^+^ and Δ*w*^−^ determine the amplitude of the variable instantaneous increases and decreases; *V_mem_*(*t_pre_*) represents the post-synaptic neuron's membrane potential at the time of arrival of the pre-synaptic spike, and θ_*mem*_ is a threshold term that determines whether the weight should be increased or decreased; the term *Ca*(*t_pre_*) represents the post-synaptic neuron's Calcium concentration, which is proportional to the neuron's recent spiking activity, at the time of the pre-synaptic spike, while the terms θ_1_, θ_2_, and θ_3_ are three thresholds that determine in which conditions the weights are allowed to be increased, decreased, or should not be updated. These “stop-learning” conditions are useful for normalizing the weights of all synapses afferent to the same neuron. They have been shown to be effective in extending the memory lifetime of recurrent spiking neural networks, and in increasing their capacity (Senn and Fusi, 2005).

In parallel to the instantaneous weight updates, the internal variable of the synapse *w_i_* is constantly being driven toward one of two stable states, depending whether it is above or below a given threshold θ*_w_*:

(2) $$\{\begin{array}{ll} \frac{d}{dt}w_{i}=+C_{drift} & \text{if }w_{i}>\theta_{w}\text{ and }w_{i}<w_{max}\\ \frac{d}{dt}w_{i}=-C_{drift} & \text{if }w_{i}<\theta_{w}\text{ and }w_{i}>w_{min} \end{array}$$

where *C_drift_* represents the rate at which the synapse is driven to its bounds, and *w_max_* and *w_min_* represent the high and low bounds, respectively. The actual weight *J_i_* of the synapse *i* is a thresholded version of the internal variable *w_i_* that is used to produce the Excitatory Post-Synaptic Current (EPSC) upon the arrival of the pre-synaptic spike:

(3) $$J_{i}=J_{max}f(w_{i},\theta_{J})$$

where *f*(*x*, θ_*J*_) can be a sigmoidal or hard-threshold function with threshold θ_*J*_, and *J_max_* is the maximum synaptic efficacy.

We will show in Section 2.2.3 experimental results that demonstrate how the circuits integrated in the ROLLS neuromorphic processor chip faithfully implement this learning algorithm.

### 2.2. The neuromorphic processor building blocks

Here we present the main building blocks used in the ROLLS neuromorphic processor chip, describing the circuit schematics and explaining their behavior.

#### 2.2.1. The silicon neuron block

**Figure 3:**
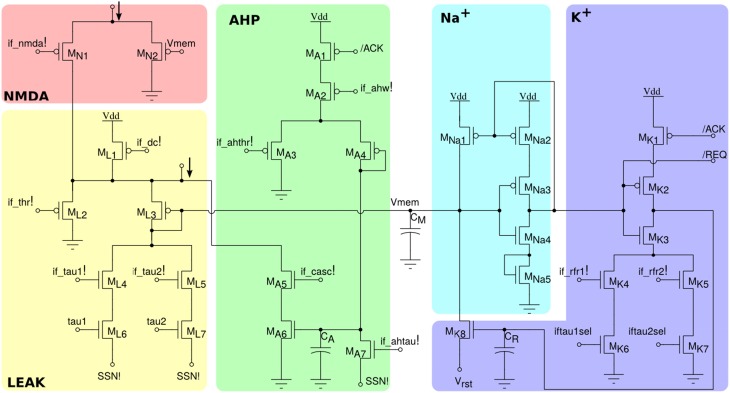
**Silicon neuron schematics**. The NMDA block implements a voltage gating mechanism; the LEAK block models the neuron's leak conductance; the spike-frequency adaptation block AHP models the after-hyper-polarizing current effect; the positive-feedback block Na^+^ models the effect of the Sodium activation and inactivation channels; reset block K^+^ models the Potassium conductance functionality.

The neuron circuit integrated in this chip is derived from the adaptive exponential I&F circuit proposed in Indiveri et al. (2011), which can exhibit a wide range of neural behaviors, such as spike-frequency adaptation properties, refractory period mechanism and adjustable spiking threshold mechanism. The circuit schematic is shown in Figure 3. It comprises an NMDA block (*M*_*N*1,*N*2_), which implements the NMDA voltage gating function, a LEAK DPI circuit (*M*_*L*1−*L*7_) which models the neuron's leak conductance, an AHP DPI circuit (*M*_*A*1−*A*7_) in negative feedback mode, which implements a spike-frequency adaptation behavior, an Na^+^ positive feedback block (*M*_*Na*1−*Na*5_) which models the effect of Sodium activation and inactivation channels for producing the spike, and a K^+^ block (*M*_*K*1−*K*7_) which models the effect of the Potassium conductance, resetting the neuron and implementing a refractory period mechanism. The negative feedback mechanism of the AHP block, and the tunable reset potential of the K^+^ block introduce two extra variables in the dynamic equation of the neuron that can endow it with a wide variety of dynamical behaviors (Izhikevich, 2003). As the neuron circuit equations are essentially the same of the adaptive I&F neuron model, we refer to the work of Brette and Gerstner (2005) for an extensive analysis of the repertoire of behaviors that this neuron model can reproduce, in comparison to, e.g., the Izhikevich neuron model.

All voltage bias variables in Figure 3 ending with an exclamation mark represent global tunable parameters which can be precisely set by the on chip Bias Generator (BG). There are a total of 13 tunable parameters, which provide the user with high flexibility for configuring all neurons to produce different sets of behaviors. In addition, by setting the appropriate bits of the relative latches in each neuron, it is possible to configure two different leak time constants (if_tau1!/if_tau2!) and refractory period settings (if_rfr1!/if_rfr2!). This gives the user the opportunity to model up to four different types/populations of neurons within the same chip, that have different leak conductances and/or refractory periods.

**Figure 4:**
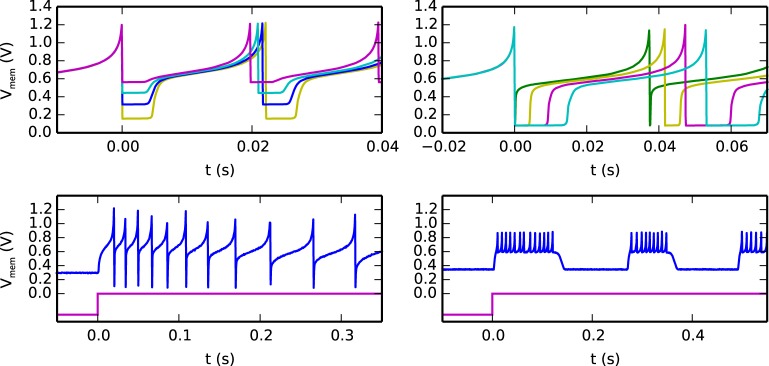
**Different biologically plausible neuron's behaviors: (top-left) membrane potential with tunable reset potential (different colors represent different reset potential settings), (top-right) membrane potential with tunable refractory period duration (different colors represent different refractory period settings), (bottom-left) neuron's spike-frequency adaptation behavior: the top trace represents the membrane potential and the bottom one represents the input current, (bottom-right) neuron's bursting behavior**.

An example of the possible behaviors that can be expressed by the silicon neuron are shown in Figure 4. The top-left quadrant shows measured data from the chip representing the neuron membrane potential in response to a constant current injection for different values of reset voltage. The top-right quadrant shows the neuron response to a constant current injection for different settings of its refractory period. The bottom-left quadrant demonstrates the spike-frequency adaptation behavior, obtained by appropriately tuning the relevant parameters in the AHP block of Figure 3 and stimulating the neuron with a constant injection current. By further increasing the gain of the AHP negative feedback block the neuron can produce bursting behavior (see bottom-right quadrant of Figure 4).

**Figure 5:**
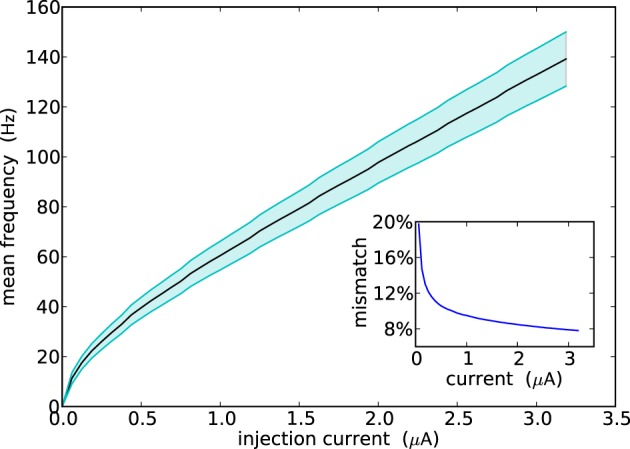
**Population response of all neurons in the array to constant injection currents**. The variance in the measurements is due to device mismatch effects in the analog circuits.

Figure 5 shows the F-I curve of all neurons in the ROLLS neuromorphic processor (i.e., their firing rate as a function of the input injection current). The plot shows their average firing rate in solid line, and their standard deviation in the shaded area. The overall mismatch in the circuit, responsible for these deviations, is extremely small, if compared to other analog VLSI implementations of neural systems (Indiveri et al., 2006; Petrovici et al., 2014; Schmuker et al., 2014). The average value obtained from the measurement results of Figure 5 is only 9.4%. The reason for this improvement lies in the increased size of some critical transistors in the soma circuit—major contributor to neuron's mismatch. For example, the *M*_*L*4_ and *M*_*L*5_ Field-Effect Transistors (FETs) that set the neuron's leak time constants are of (W/L) size of (2 μm/4 μm), while *M*_*Na*3_ and *M*_*Na*4_, responsible for the firing threshold are of size (4 μm/0.4 μm) and (1 μm/4 μm), respectively.

**Figure 6:**
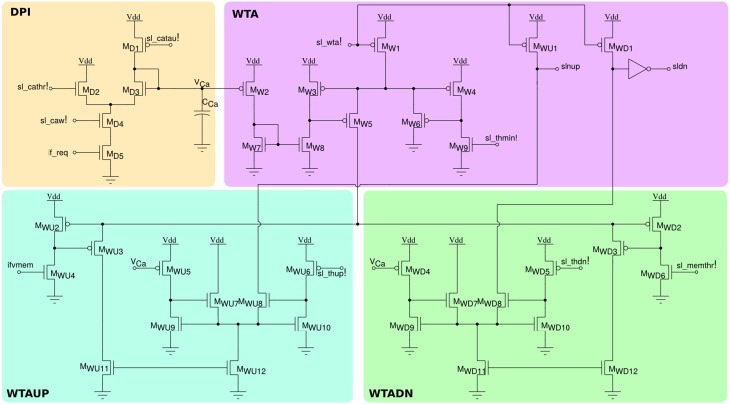
**Post-synaptic learning circuits for evaluating the algorithm's weight update and “stop-learning” conditions**. The DPI circuit *M*_*D*1−5_ integrates the post-synaptic neuron spikes and produces a current proportional to the neuron's Calcium concentration. Three current-mode winner-take-all circuits *WTA*, *WTAUP*, and *WTADN* compare the Calcium concentration current to three set thresholds sl_thmin!, sl_thdn!, and sl_thup!, while the neuron's membrane current is compared to the threshold sl_memthr!.

In addition to the neuron soma circuit, this block contains also post-synaptic plasticity circuits that are necessary for evaluating the weight update and “stop-learning” conditions described in Section 2.1.2. In particular these circuits integrate the spikes produced by the neuron into a current that models the neuron's Calcium concentration, and compare this current to three threshold currents that correspond to θ_1_, θ_2_, and θ_3_ of Equation (1). In parallel, the neuron's membrane current (which is equivalent to the membrane potential in the theoretical model) is compared to an additional threshold equivalent to θ_*mem*_ of Equation (1). The schematic diagram of this circuit is shown in Figure 6. The post-synaptic neuron's Calcium concentration is computed using the DPI *M*_*D*1−*D*5_; the comparisons with the fixed thresholds are made using three current-mode Winner-Take-All (WTA) circuits *M*_*W*1−*W*9_, *M*_*WU*1−*WU*12_, and *M*_*WD*1−*WD*12_. The digital outcomes of these comparisons set the signals slnup and sldn which are then buffered and transmitted in parallel to all synapses afferent to this neuron belonging to the long-term plasticity array.

#### 2.2.2. The long-term plasticity synapse array

**Figure 7:**
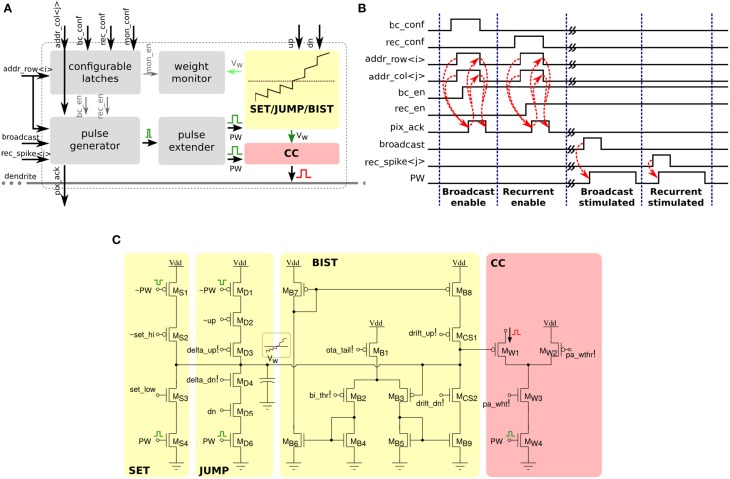
**Long-term plasticity synapse array element**. **(A)** Plastic synapse configuration logic block diagram. **(B)** Timing diagram for broadcast and recurrent activation modes in one synapse using 4-phase handshaking protocol. Dashed red lines show the sequence between signals. **(C)** Schematic diagram of the bi-stable weight update and current generator blocks.

Each of the 256×256 synapse circuits in the long-term plasticity array comprises event-based programmable logic circuits for configuring both synapse and network properties, as well as analog/digital circuits for implementing the learning algorithm of Section 2.1.2. Figure 7 shows both digital and analog circuit blocks. The digital logic part, shown in Figure 7A has an pulse generator circuit that manages the handshaking signals required by the AER protocol, and three one-bit configurable latches: one latch sets/resets the MON_EN signal, which enables/disables the synapse monitor circuit, which buffers the synapse weight *V_w_* signal for off-chip reading. The remaining two latches are used to set the BC_EN and REC_EN signals, which control the activation modes of the synapse. There are three different activation modes can be configured: direct activation, broadcast activation and recurrent activation. Figure 7B shows a timing diagram in which the relative latches for enabling broadcast and recurrent activation modes are configured in a synapse, using a 4-phase handshaking protocol. In the direct activation mode the synapse is stimulated by an AER event that has the matching row and column address. In the broadcast activation mode the synapse is stimulated by an AER broadcast event (that has a dedicated address word) which targets the matching column address. All synapses belonging to the same column that have the BC_EN bit set high get stimulated in parallel, when the matching broadcast event is received. In the recurrent activation mode the synapse of column *j* is stimulated when the on-chip post-synaptic neuron of row *j* spikes. Therefore, it is possible to connect, internally, neuron *i* to neuron *j* by setting the REC_EN bit high of the synapse in row *i* and column *j*. In addition to these circuits, there is a pulse extender circuit which can increase the duration of the input pulse from nano-seconds to hundreds of micro-seconds.

The schematic diagram of the analog/digital weight update circuits is shown in Figure 7C. These circuits are subdivided into four sub-blocks: the SET block can be used to set/reset the bistable state of the synaptic weight by sending an AER event with the matching address and properly asserting the configuration signals set_hi and set_low. The JUMP block increases or decreases the synaptic weight internal variable (i.e., the voltage *V_w_*) depending on the digital signals up and dn, that are buffered copies of the ones generated in the silicon neuron stop-learning block (see Section 2.2.1). The heights of the up and down jumps can be set by changing the delta_up! and delta_dn! signals. The BIST block consists of a wide-range transconductance amplifier configured in positive feedback mode, to constantly compare the *V_w_* node with the threshold bi_thr!: if *V_w_* > bi_thr! then the amplifier slowly drives the *V_w_* node, drifting toward the positive rail, otherwise it actively drives it toward the ground. The drift rates to the two states can be tuned by biases drift_up! and drift_dn!, respectively. The current converter (CC) block converts the *V_w_* voltage into a thresholded EPSC with maximum amplitude set by pa_wht!.

**Figure 8:**
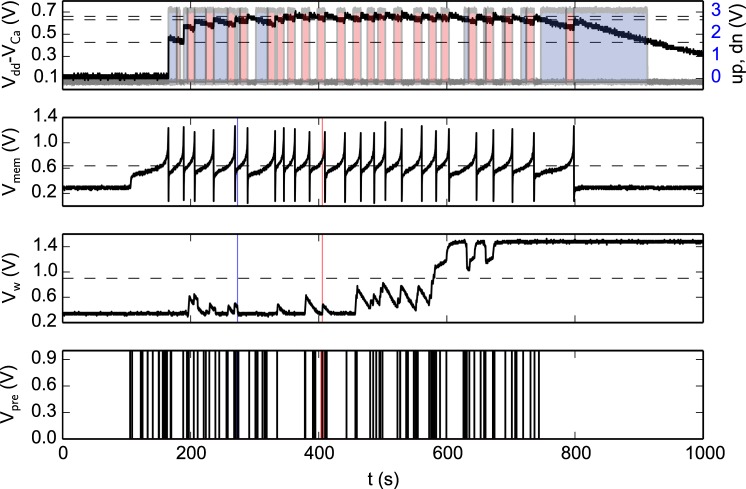
**Spike-based learning circuit measurements**. The bottom trace represents the pre-synaptic input spikes; the second trace from the bottom represents the bi-stable internal variable (node V_*w*_ of Figure 7); the third trace represents the post-synaptic neuron's membrane potential; and the top trace shows both a voltage trace proportional to the neuron's integrated spiking activity as well as the digital control signals that determine whether to increase (red shaded area), decrease (blue shaded area) or leave V_*w*_ unchanged (no shaded area). The horizontal lines represent the thresholds used in the learning algorithm (see Section 2.1.2), while the vertical lines at *t* = 273 s (blue line) and *t* = 405 s (red line) are visual guides to show where the membrane potential is, with respect to its threshold, for down and up jumps in V_*w*_ respectively.

Figure 8 shows experimental results that highlight the features of both synapse and neuron learning circuits in action: weight updates are triggered when the pre-synaptic spikes arrive, and when the post-synaptic neuron's Calcium concentration is in the appropriate range. Depending on the value of the Calcium concentration signal, the digital up and dn signal turn on or off. The weight internal variable is increased or decreased depending on where the membrane potential is with respect to the membrane threshold (see highlighted weight updates at *t* = 273 and *t* = 405). This variable is actively driven to the low or high bounds, depending if it is below or above the weight hreshold.

#### 2.2.3. The short-term plasticity synaptic array

**Figure 9:**
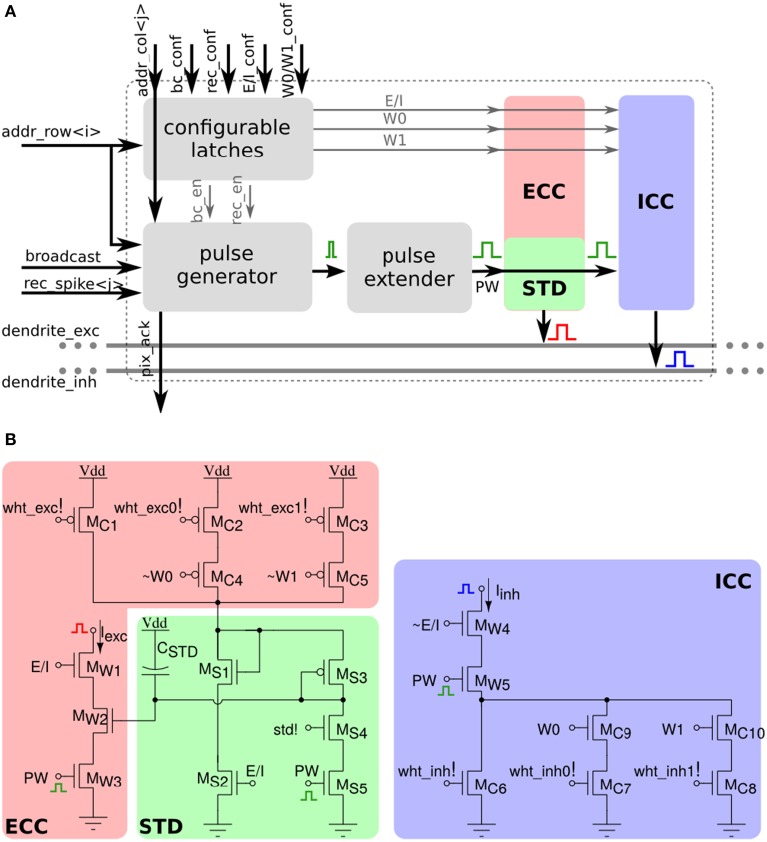
**Short-term plasticity synapse array element. (A)** Block diagram of the synapse element. **(B)** Transistor level schematic diagram of the excitatory and inhibitory pulse-to-current converters.

The array of STP synapses contains circuits that allow users to program the synaptic weights, rather than changing them with a fixed on-chip learning algorithm. Specifically, each synapse has a two-bit programmable latch that can be used to set one of four possible weight values. In addition, it has an extra latch that can set the type of synapse (excitatory or inhibitory). In the excitatory mode, the synapse has additional circuits for modeling Short-Term Depression (STD) dynamics (Rasche and Hahnloser, 2001; Boegerhausen et al., 2003) whereby the magnitude of the EPSC decreases with every input spike, and recovers slowly in absence of inputs. Figure 9 shows both a block diagram of all synapse components, and the schematic diagram of the synapse analog circuits. In addition to the latches for setting the weight, there are two extra latches for configuring the synapse activation mode. As for the long-term-plasticity synapses, there are three possible activation modes: direct, broadcast, and recurrent (see Section 2.2.2).

The left panel of Figure 9B shows the excitatory CC and the STD circuit. The CC at the top generates a current that is proportional to the 2-bit weight. The proportionality constant is controlled through analog biases. This current charges up the *C_STD_* capacitor through the diode connected p-FET *M*_*S*3_ so that at steady state, the gate voltages of *M*_*S*1_ and *M*_*W*2_ are equal. A pre-synaptic pulse on the *PW* port activates the *I_exc_* current branch, and produces a current that initially is proportional to the 2-bit weight original current. At the same time, the *PW* pulse activates also the STD branch through transistor *M*_*S*5_ and an amount of positive charge that is controlled by the bias *STD* is removed from the capacitor *C_STD_*. The gate voltage of *M*_*W*2_ is now momentarily lower than that of *M*_*S*1_, and recovers slowly through the diode connected p-FET *M*_*S*3_. Pulses that arrive before the capacitor voltage has recovered completely will generate a current that is smaller than the original one, and will further depress the effective synaptic weight through the STD branch. The excitatory block is only active if the *E*/*I* voltage is high. If *E*/*I* is low, the inhibitory current DAC in the right panel of Figure 9B is active and generates a weight-proportional inhibitory current on *PW* pulses.

**Figure 10:**
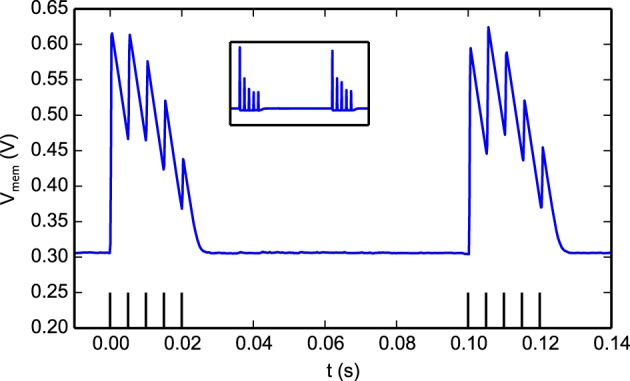
**The effect of short-term depression on EPSC magnitudes**. Two bursts separated by 100 ms were sent to a programmable synapse. Each burst has 5 spikes with an inter-spike interval of 5 ms. Within a burst, The jumps in the neuron *V_mem_* gradually get smaller as the synapse is depressed and the magnitude of the EPSCs it generates decreases. After the first burst, the synapse efficacy recovers as can be seen in the response to the second burst. The figure inset shows the derivative of the membrane potential which is equivalent to the synaptic EPSCs (minus the neuron leak).

Figure 10 illustrates how the STD behavior in the synapse: a spike burst was used to activate a programmable synapse. This resulted in a drop in synaptic efficacy during the later part of the burst. During a period of no stimulation the synapse recovered and responded with large Excitatory Post-Synaptic Potentials (EPSPs) to the initial part of the following burst, before depressing again. The responses to the two bursts are not identical in Figure 10 as the state of the neuron, synapse, and DPI circuits are not exactly the same at the onset of each burst.

#### 2.2.4. The peripheral input/output blocks

The peripheral digital circuits are used to transmit signals into and out of the chip. Given the real-time nature of our system, we use asynchronous digital circuits and quasi-delay-insensitive circuit design techniques (Manohar, 2006) to avoid discretization or virtualization of time. The AER communication protocol used encodes signals as the address of the destination synapse or as a control word for the input side, and as the address of the sender neuron in the output circuits.

##### 2.2.4.1. AER input circuits

Input spike events as well as chip configuration events are sent through a common input interface that uses a 21-bit address space. Input addresses are decoded into a total of 1,249,553 possible patterns subdivided into three categories: *Addressing*, *Local configuration*, and *Global configuration*. *Addressing* inputs are decoded into a row and column address and are interpreted as a spike Address-Event (AE), which are sent to the desired target synapse of a target neuron. *Local configuration* AEs contain the row and column address of the target element as well as extra configuration bits that are written to the local latches of the addressed element. *Local configuration* patterns include commands for setting the type of synapse, programming its weight, or enabling broadcast or recurrent connections. Finally, the *Global configuration* inputs are decoded into configuration signals that represent global variables, stored onto registers in the periphery (rather than within the synapse or neuron elements). For example, the signals used to set the state of the synapse de-multiplexer are *Global configuration* signals. See the Supplementary Material for additional details on these circuits.

##### 2.2.4.2. AER output

Each of the 256 neurons is assigned an 8-bit address for the output bus. When a neuron spikes, its address is instantaneously sent to the output AER circuits using the common four-phase handshaking scheme. Although neurons operate in a fully parallel fashion, their AEs can only access the shared output bus in a serial fashion. To manage possible simultaneous spike collisions the output AER circuits include an arbiter circuit that only grants access to the external bus to one neuron at a time. Details of these circuits are provided in the Supplementary Material.

## 3. Results

Here we demonstrate the capabilities of the ROLLS neuromorphic processor device with examples of hardware emulation of computational neuroscience models and pattern recognition in a machine vision task.

### 3.1. Attractor networks

In this experiment we explored the collective dynamics of multiple populations of spiking silicon neurons that emulate the biophysics of cortical neurons organized in attractor networks (Amit, 1992). These types of networks are considered a basic computational primitive of neural processing systems. Their ability to exhibit self sustained activity is thought to be one of the basic requirements for exhibiting multiple types of cognitive processes and functions. Their collective dynamics represents the neural correlates of processes involved in working memory, perceptual decision making and attention.

**Figure 11:**
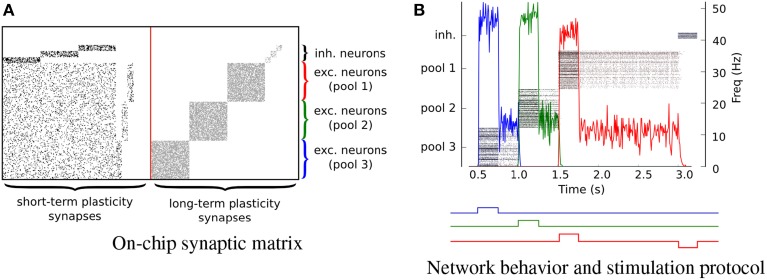
**Attractor networks**. Three clustered pools of 64 neurons are configured as attractor networks. **(A)** The on-chip synaptic matrices in which every dot represents a connected synapse. **(B)** The output spiking activity of the network. Every pool of neuron is a competing working memory. The timing of the inputs is shown with square waves under the plot. Different colors represent different inputs. The down jump of the red line represents the stimulus to the inhibitory pool of neuron responsible for switching off the on-chip memory.

We implemented the hardware attractor networks following the theories and methods proposed in Amit (1992); Wang (1999); Amit and Mongillo (2003); Del Giudice et al. (2003); Giulioni et al. (2012). We constructed an architecture comprising six pools of neurons recurrently connected. Specifically, there are three pools of 64 excitatory neurons and three pools of 10 inhibitory neurons. Neurons in each pool receive local excitation via recurrent connections implemented via the on-chip long-term synaptic plasticity circuits. In Figure 11 each point represents a synaptic contact (i.e., an active synapse in the corresponding STP or LTP synaptic matrix). The recurrent connectivity via the LTP synapses is set to have a probability of 70% for the excitatory connections and 40% for the inhibitory ones, i.e., they have connectivity parameters *c^e^_ee_* = 0.7, *c^e^_ii_* = 0.4, respectively (see dots in Figure 11A). We further configured the connectivity matrix of the STP synapses such that every excitatory pools of neurons is homogeneously connected with all other excitatory pools with excitatory connectivity parameter *c^e^_ee_* = 0.2 and inhibitory connectivity parameter *c^i^_ee_* = 0.2. Inhibitory pools of neurons are connected to their corresponding excitatory pools (e.g., inhibitory pool #1 is connected to excitatory pool #1) via inhibitory synapses, with a connectivity parameter *c^i^_ei_* = 0.4. Excitatory pools of neurons are connected to their respective inhibitory pools of neurons via the STP excitatory synapses, with a connectivity parameter *c^e^_ie_* = 0.7. The behavior of the network when stimulated by a external transient stimuli is shown in Figure 11B. The profile of the external stimuli is depicted by the square waves below the plot of Figure 11B. The different colors indicate inputs to the different corresponding populations. The input stimuli are a series of Poisson spike trains, generated artificially and sent via the AER protocol to the chip virtual synapses. The mean rate of the input spike trains is *v_in_* = 100 *Hz* and their duration is *t* = 0.5 *s*. When the attractor networks are being driven by external stimuli their activity reaches a mean rate of approximately 50 Hz and, after the removal of these stimuli, the pools of neurons relax to a sustained state of activity of about 15 Hz, indicating that the neurons settled into their attractor states. This persistent activity is the neural correlate of working memory and can be exploited as an asynchronous distributed memory state that has peculiar dynamical properties of error correction, pattern completion, and stability against distractors (Amit, 1992).

If a population is in an attractor state, a transient stimulus to a different pool of neurons shuts down its activity via direct inhibitory connections (on the STP synaptic matrix), and brings the newly stimulated pool of neurons into a new attractor state. If we inhibit an active pool of neurons directly, with an external stimulus the population is reset and becomes inactive. This is evident in Figure 11B at *t* = 3 s, when a Poisson stimulus of mean rate ν = 200 Hz is used to inhibit all attractor networks. This experiment demonstrates how it is possible to implement robust state dependent computation and reliable memory storage using sets of 64 slow and imprecise silicon neurons. A similar, but more elaborate experiment showing how these types of circuits can be used to synthesize context-dependent behavior in neuromorphic agents, in the context of cognitive computation was recently presented in Neftci et al. (2013), using the same types of circuits and principles. The implementation of plausible neural collective dynamics in neuromorphic substrates is an important step also for future nano-technologies that are likely to be affected by device mismatch and unreliability characteristics.

### 3.2. Multi-perceptron network

**Figure 12:**
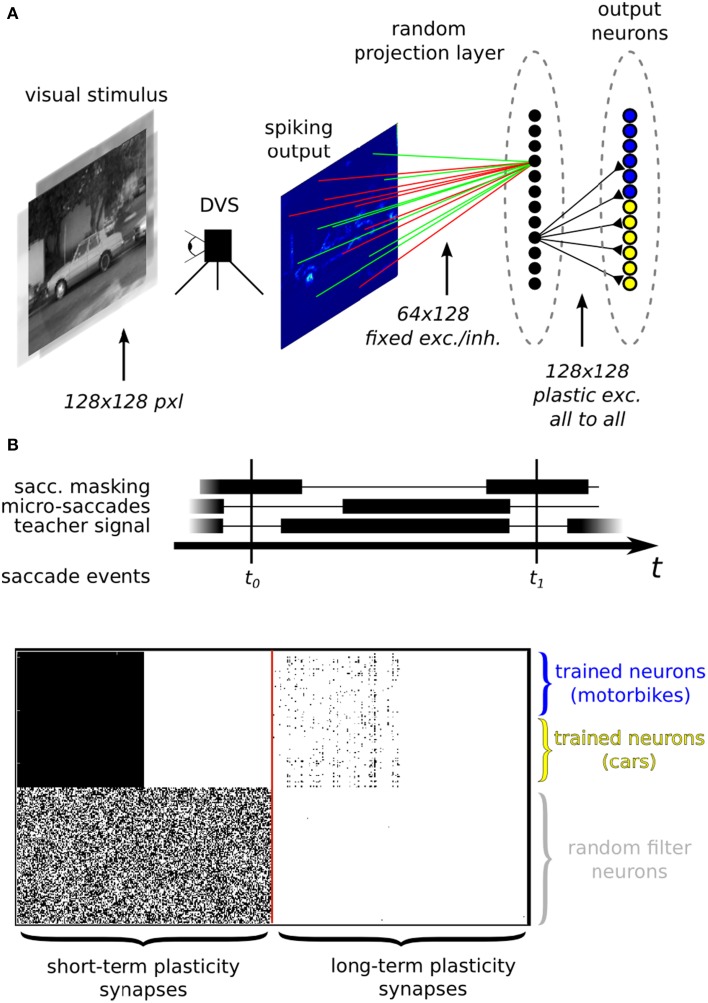
**(A)** Image classification example using inputs from a DVS. **(A)** Top: neural network architecture. Two different classes of images (here motorbikes or cars) are displayed on a screen with a small jitter applied at 10 Hz. A random subset of the spikes emitted by the DVS are mapped to 128 hidden layer neurons. Specifically, each of the 128 neurons is connected to 64 randomly selected pixels with either positive or negative weights, also set at random. The output neurons in the last layer receive spikes from all the 128 hidden layer neurons, via plastic synapses. The output layer neurons are also driven by an external “teacher” signal which is correlated with one of the image classes. **(A)** Bottom: diagram of the experimental protocol timeline. Notice the presence of a saccade inhibition mechanism which electronically suppresses DVS input during a virtual saccade, i.e., when the displayed image is replaced with the next one. **(B)** Synaptic matrices of the ROLLS neuromorphic processor showing the hardware configuration of the classification neural network. The STP synapses represent the synapses of the hidden layer; the LTP synapses represent the synapses of the output layer.

Neuromorphic systems are an ideal electronic substrate for real-time, low-latency machine vision (Serrano-Gotarredona et al., 2008; Delbruck and Lang, 2013; O'Connor et al., 2013). Here we present a feasibility study which demonstrates how the ROLLS neuromorphic processor can be used in conjunction with a spiking vision sensor for learning to solve an image classification task. In this experiment (see Figure 12), we used a DVS, interfaced to our chip via a commercially available digital board, used to route signals from the vision sensor to the chip. We implemented a two-layer spiking neural network which processes the visual stimuli by extracting sparse random features in real-time. The network is composed of 128 VLSI hidden neurons and 128 VLSI output neurons on the ROLLS neuromorphic processor. We trained 64 of the VLSI output neurons of the network to become selective to one of two image classes, and the other 64 to become selective to the other class, via supervised learning protocol.

The experimental protocol consists of showing a sequence of static images of objects from the Caltech 101 dataset coupled with a teacher signal to steer the activity of the output neurons. The DVS is put in front of a screen where the images are displayed. During the presentation, the images are flashed with a small jitter around the center of the visual field to simulate microsaccadic eye movements. The movement causes the DVS retina to continuously stream spike trains corresponding to the edges of the objects in the image. The spike trains are then routed to the STP synapse array, stimulating a population of neurons corresponding to the hidden layer of the neural network. The spikes from the hidden layer neurons are internally routed to the LTP plastic synapse array, thus activating the neurons of the output layer. With every training image, a corresponding teacher signal is provided to one of the two subgroups of the output layer neurons, depending on the image class, to associate stimulus with class. To remove artifacts generated during the transition from one presentation of an image to the next, we gated the DVS spikes, simulating a saccadic suppression mechanism analogous to the one observed in biology (Ross et al., 1996).

The performance of this experiment strongly depends on the right choice of parameters for the neural and synaptic dynamics. For this particular demonstration we chose to disable most of the complex aspects of the neural dynamics and optimized neuron and synapse parameters to obtain reasonable activity patterns in the hidden layer neurons. The activity in this layer is indeed the most important since it drives the plastic synapses that belong to the output layer neurons.

After training, our classification system was able to respond selectively to natural images of cars and motorbikes taken from the Caltech 101 database. Although an extensive characterization of the system's ability to perform object recognition is out of the scope of this work, we draw the following conclusions from our experiment:

- The choice of fixed, random projections from the input layer was surprisingly effective, though certainly not optimal for the task at hand.
- A better solution would be to include an unsupervised learning stage in the training protocol to optimize the weights of the convolution layer as in traditional machine learning approaches (LeCun et al., 1998; Le et al., 2012) and in neural systems (Olshausen and Field, 1997; Masquelier et al., 2009; Nessler et al., 2009). However, this stage would require the presentation of a large number of patterns and sophisticated synaptic plasticity rules.

**Figure 13:**
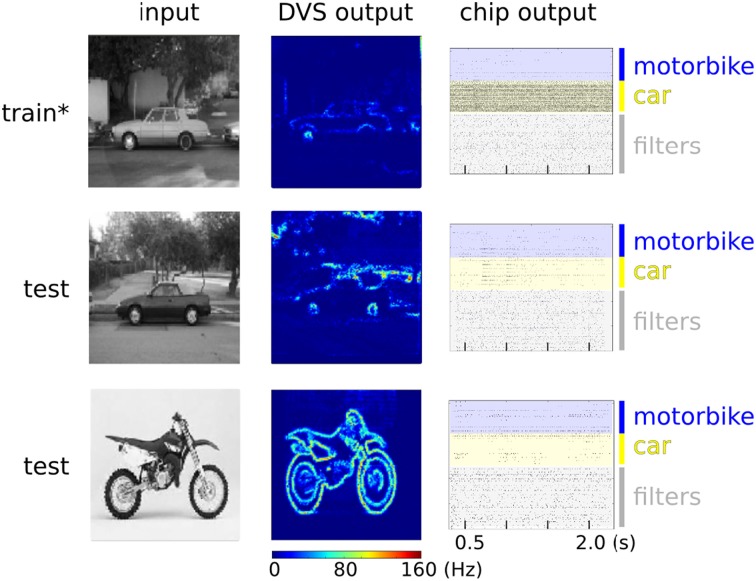
**Spiking activity of the hardware neurons during the training (upper panel) and testing phase (middle and lower panels)**. **Left column**: examples of raw images from the Caltech 101 database. **Middle column**: heat map of the DVS spiking activity, where each pixel color represents the pixel's mean firing rate. **Right column**: raster plots of the ROLLS neuromorphic processor neurons. The star on the top panel label indicates that during the training phase an additional excitatory “teacher” signal was used to stimulate the “car” output neurons to induce plasticity. During testing no teacher signal is provided and only the excitatory currents from the input synapses drive the classifier activities. The average firing rates of the output layer for “motorbike” and “car” neuron pools are 6.0 Hz and 80.9 Hz during training. During testing with a “car” image they are 7.1 Hz and 11.1 Hz. During testing with a “motorbike” image they are 7.4 Hz and 4.9 Hz.

Our network of randomly connected neurons projects the input stimuli into a high-dimensional space where they can be classified by linear models but with far less parameter optimization (Barak and Rigotti, 2011). This strategy is related to some of the state-of-the-art machine learning algorithms for pattern classifications, such as Support Vector Machines (SVMs) (Vapnik, 1995). Clearly, the generalization properties of our system are not comparable to standard machine learning approaches but they are also expected to scale with the number of randomly connected neurons in the hidden layer (Rigotti et al., 2010; Barak et al., 2013). Notice also that we haven't exploited any temporal structure of the input data, though we recently demonstrated that our hardware supports this functionality (Sheik et al., 2012a,b, 2013). For cases in which the temporal structure of the input stimuli is relevant, it would be possible to follow alternative approaches, for example by interconnecting the neurons in the hidden layer to form a Liquid State Machine (LSM) (Maass et al., 2002). This solution would be particularly interesting in situations where information hidden in the fine temporal structure is expected to impact the performance of the recognition system. Also for this approach, it would be sufficient to provide an output layer analogous to the one used in our experiment, that could be trained in an analogous way. In our example we used multiple neurons clustered into two distinct pools in the output layer for our simple two-class discrimination problem, (e.g., instead of using just two output neuron units). The rationale behind this choice is that, given the many sources of noise in the system (the micro-saccadic movements, the DVS spiking output, the stochastic plasticity mechanism, the hardware mismatch), each neuron taken singularly is not expected to perform well on the task (i.e., it will implement a “weak” classifier, showing low class specificity). However, the performance of the overall system improves as responses aggregated from multiple neurons are considered. This can be visually appreciated from the raster plots of Figure 13 where only population-level firing rates are selective for the input classes, but not the single neuron activities. This phenomenon is directly related to a notorious machine learning technique that uses “boosting” to improve the performance of weak-classifiers (Breiman, 2001; Schapire and Freund, 2012).

## 4. Discussion

Unlike conventional von Neumann processors that carry out bit-precise processing and access and store data in a physically separate memory block, the ROLLS neuromorphic processor uses elements in which memory and computation are co-localized. The computing paradigm implemented by these types of neuromorphic processors does not allow for the virtualization of time, with the transfer of partial results back and forth between the computing units and physically separate memory banks at high speeds. Instead, their synapse and neuron circuits process input spikes on demand as they arrive, and produce their output responses in real-time. Consequently, the time constants of the synapses and neurons present in these devices need to be well-matched to the signals the system is designed to process. For the case of real-time behaving systems that must interact with the environment, while processing natural signals in real-time, these time constants turn out to be compatible with the biologically plausible ones that we designed into the ROLLS neuromorphic processor. As we implemented non-linear operations in each synapse (such as short-term depression or long-term plasticity), it is not possible to time-multiplex linear circuits to reduce the area occupied by the synaptic matrix array. As a consequence, our device is essentially a large memory chip with dedicated circuits for each synapse that act both as memory elements and computing ones. This approach is complementary to other recent ones that focus on accelerated neural simulations (Bruederle et al., 2011), or that target the real-time emulation of large populations of neurons but with no on-chip learning or adaptive behaviors at the synapse level (Benjamin et al., 2014).

The device we describe here is ideal for processing sensory signals produced by neuromorphic sensors (Liu and Delbruck, 2010) and building autonomous behaving agents. The system level examples demonstrated in Section 3 show how this can be achieved in practice: the hardware attractor network experiment focuses on the idea that the functional units of the cortex are subset of neurons that are repeatedly active together and shows that such units have the capability of storing state-dependent information; the pattern classification experiment demonstrates how it is possible to implement relatively complex sensory processing tasks using event-based neuromorphic sensors.

Our results demonstrate the high-degree of programmability of our device as well as its usability in typical application domains. Its properties make it an ideal tool for exploring computational principles of spiking systems consisting of both spiking sensors and cortical-like processing units. This type of tools are an essential resource for understanding how to leverage the physical properties of the electronic substrate as well as the most robust theories of neural computation in light of the design of a new generation of cortex-like processors for real-world applications. The multi-chip system is supported by the use of a newly developed software front-end, PyNCS, which allows rapid integration of heterogeneous spiking neuromorphic devices in unique hardware infrastructure and continuous online monitoring and interaction with the system during execution (Stefanini et al., 2014). In order to integrate the DVS and ROLLS in the existing software and hardware infrastructure, it was necessary to list the address specifications for the spiking events and for the configuration events in Neuromorphic Hardware Mark-up Language (NHML) files, the neuromorphic mark-up language used by PyNCS to control the neuromorphic system.

The potential of the approach proposed in this work for building intelligent autonomous systems is extremely high, as we develop brain-inspired computing devices embedded with learning capabilities that can interact with the environment in real time. Substantial progress has already been made in the theoretical domain (Schöner, 2007; Rutishauser and Douglas, 2009), and preliminary results have already been demonstrated also with neuromorphic cognitive systems (Neftci et al., 2013) synthesized by the user. The ROLLS neuromorphic processor described in this work can therefore contribute to extending the current state-of-the-art by providing also adaptation and learning mechanisms that could allow these systems to learn the appropriate network properties to implement autonomous cognitive systems.

## 5. Conclusions

We presented a mixed-signal analog/digital VLSI device for implementing on-line learning spiking neural network architectures with biophysically realistic neuromorphic circuits such as STP synapses, LTP synapses and low-power, low-mismatch adaptive I&F silicon neurons. The proposed architecture exploits digital configuration latches in each synapse and neuron element to guarantee a highly flexible infrastructure for programming, with the same device, diverse spiking neural network architectures.

All the operations of the chip are achieved via asynchronous AE streams. These operations include sending events to the chip, configuring the topology of the neuron network, probing internal variables, as well as programming internal properties of synapse and neurons. The parameters for different synapse and neuron behaviors can be fine tuned by programming the temperature-compensated on-chip BG.

The ROLLS neuromorphic processor can be used to carry out basic research in computational neuroscience and can be exploited for developing application solutions for practical tasks. In particular, this architecture has been developed to study spike-based adaptation and plasticity mechanism and to use its ability to carry out on-chip on-line learning for solving tasks that require the system to adapt to the changes in its input signals and in the environment it interacts with.

### Conflict of interest statement

The authors declare that the research was conducted in the absence of any commercial or financial relationships that could be construed as a potential conflict of interest.
