# Supplementary material for: A reconfigurable on-line learning spiking neuromorphic processor comprising 256 neurons and 128K synapses
Source: Front Neurosci. 2015 Apr 29;9:141. doi: 10.3389/fnins.2015.00141 (PMC4413675; doi:10.3389/fnins.2015.00141)
Supplement: Supplementary file 1 [file Presentation1.PDF]

Frontiers in Neuroscience

Research Article
23 February 2015

Supplementary Material: A Reconfigurable On-line Learning Spiking Neuromorphic Processor

Ning Qiao, Hesham Mostafa, Federico Corradi, Marc Osswald, Fabio Stefanini, Dora Sumislawska, and Giacomo Indiveri

Institute of Neuroinformatics, University of Zurich and ETH Zurich, Zurich, Switzerland

Correspondence*:
Giacomo Indiveri
Institute of Neuroinformatics, University of Zurich and ETH Zurich, Winterthurerstrasse 190, 8057 Zurich, Switzerland, giacomo@ini.uzh.ch

Hardware implementation of neural computation from the interaction of different forms of plasticity

0.1 THE VIRTUAL SYNAPSE ARRAY

In this array there are two types of synapse circuits: one for representing the contribution of excitatory synapses, and one for the inhibitory synapses. Both excitatory and inhibitory synapses in this 256×2 array are designed to represent a dendritic branch that linearly sums their inputs coming from a large number of presynaptic sources, whose spikes are time multiplexed to the address line matching the synapse row address. For both excitatory and inhibitory synapses, inputs sent to them will have the same weight and the same dynamics. Both weight and synapse time-constant can be controlled through analog biases. The specific circuit that implements one of these virtual synapses is a Differential Pair Integrator (DPI). The schematic of both excitatory and inhibitory virtual synapses is shown in Fig. 1. These synapses are not part of the recurrent connectivity matrix and can only be stimulated by external Address-Event Representation (AER) events.

0.2 AER INPUT/OUTPUT CIRCUITS

0.2.1 AER Input Circuits. Figure 2a shows the schematic of the AER input circuits: the *Address decoding* block latches the input AER address bits upon a valid *pixel request* (see the PixReq signal in Fig. 2a) and decodes them into the appropriate input patterns. The *Control logic* block of Fig. 2a implements an extended version of a Mueller-Pipeline element (**J. Sparsø**, 2001) to decouple the internal *pixel request* termination from the external *chip request* (see the ChipReq signal in Fig. 2a and 2b) and therefore makes the length of the *pixel request* pulse independent from possible off-chip communication delays. This is necessary, as the duration of the *pixel request* directly affects the width of the pulse that produces the synaptic current. A full handshake transaction of the proposed input protocol is shown in Fig. 2b (solid lines) and compared to a standard 4-phase bundled-data protocol (dashed lines).

0.2.2 AER Output Figure. 3 shows a block diagram of the output AER interface. When a neuron spikes, its piplelining element immdeiately completes the 4-phase handshake with the neuron so that the neuron is reset and begins integrating input again. The stored request in the pipelining element is then forwarded to the arbiter. The arbiter selects one of the potential simultaneous requests from the pipelining elements, enqueing all other requests; when the arbiter circuits acknowledge a request from a neuron's pipelining

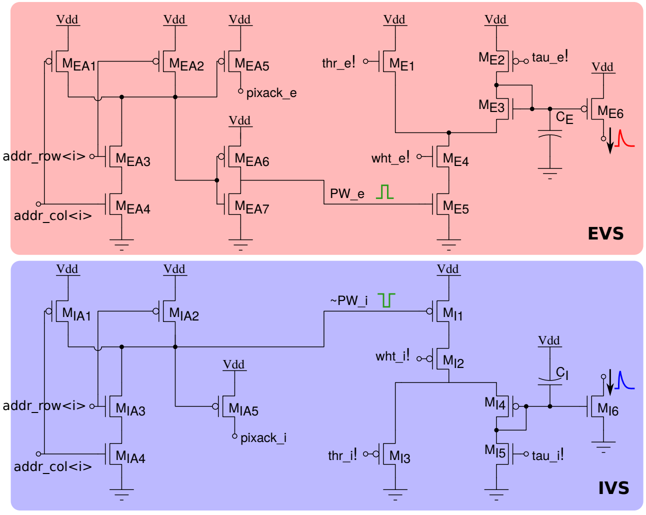

Figure 1: Top two panels: schematics of the virtual excitatory synapse and its associated DPI circuit. Bottom two panels: schematics of the virtual inhibitory synapse and its associated DPI circuit.

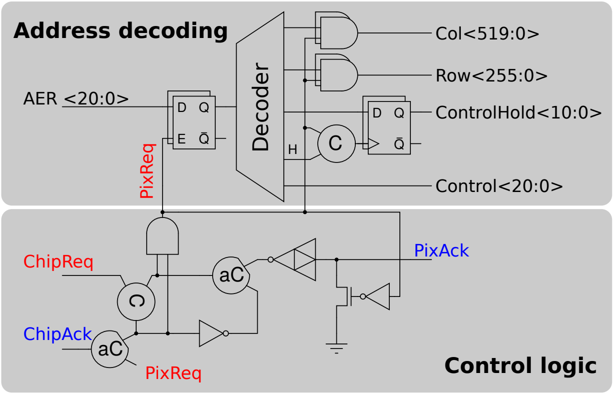

(a)

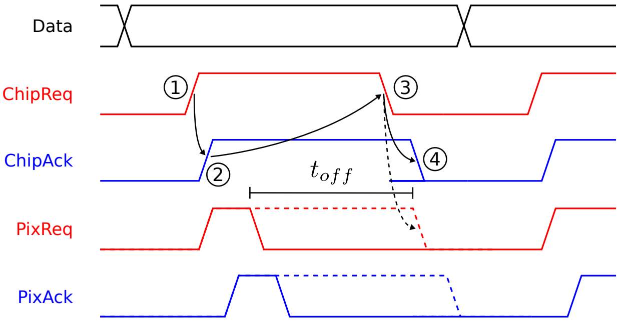

(b)

Figure 2: (a) Input schematic comprising an address decoding block and a block for the control logic. (b) Four-phase handshaking protocol for sending inputs to the chip. The dashed lines show a common four-phase bundled-data protocol while the solid lines represent the proposed modification. The *pixel request* (PixReq) terminates before the *chip request* (ChipReq) and is thus not sensitive to off-chip delays (t_{off}).

element, the encoder block encodes the address of that neuron and puts it on the output address bus; at this stage, the off-chip handshake block initiates the AER four-phase handshaking mechanism with the external receiver. Once the external handshake completes, the arbiter resets the active pipelining element by deasserting its acknowledge line. A new request from one of the pipleining elements waiting in the queue can then be processed.

While the arbitration circuits are useful to preserve and transmit all spikes generated by the neurons (at the cost of introducing small delays in case of collisions), the pipelining circuits are useful for decoupling the neuron dynamics from the potential delays and the variability produced by the interaction with off-chip

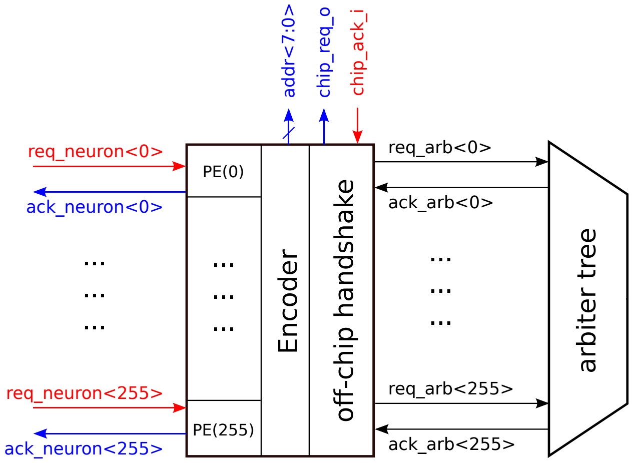

Figure 3: Block diagram of the output AER interface. See text for description.

receivers. The fully asynchronous ouput AER interface was automatically generated, down to layout level, from behavioral hardware description language (HDL) code using the methodology presented in (**?**).

0.3 ANALOG TO DIGITAL EVENT-BASED CONVERTER

The analog to digital block serves to convert a probed internal analog variables of the array of silicon neurons as membrane current, or synaptic current in digital events interfaced through the Address-Event Representation scheme. These Analog to Digital Converter (ADC)s are especially useful when working in low current regime, where dissipations in the padframe can corrupt the signal being probed. We implemented a pulse ADC with AER output circuits.

0.4 AD PULSE OUTPUT CIRCUIT

The ADC pulse-output circuit is an asynchronous clock-less circuits that is based on the same principle of the Integrate-and-Fire (I&F) neuron; it produces spikes at a rate that is proportional to the intensity of its input. The circuit schematic is shown in fig. 4a and it comprises a I&F neuron (Calib&FB), an output buffer (BUF) and two programmable branches that enable the selection of two different branches (p-type) or (n-type). The programmable input branch can be selected via the digital control signal SelN. The circuit can also be switch-off via the enable control signal En. In fig. 4b we show a reconstructed somatic current from the output this circuit. The characteristic of the AD pulse output circuit is shown in fig. 5.

0.5 EXPERIMENTS PARAMETERS

In here we report the bias current setting used in the experiments of section 4.1 and 4.2.

Table 1: Soma bias currents

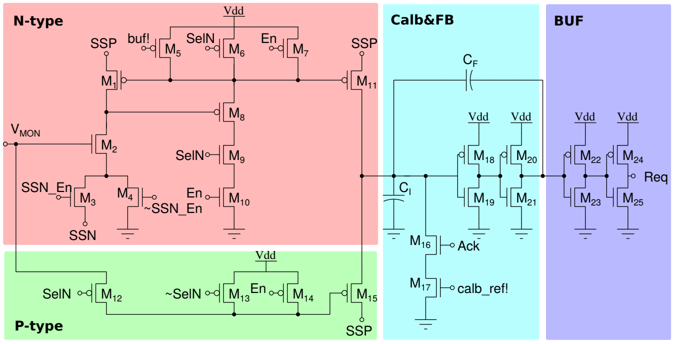

(a) AD Pulse Circuit Schematic.

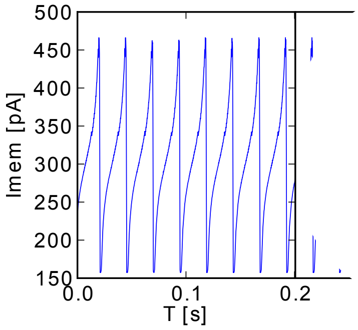

(b) Pulse Output: somatic current.

Figure 4

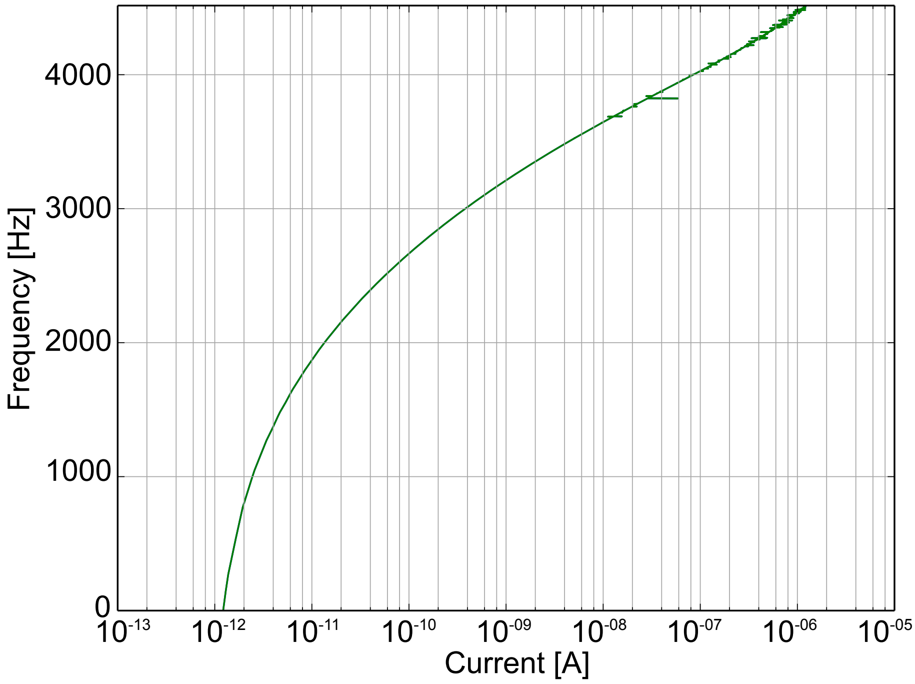

Figure 5: Current to frequency converter characteristics

Current value [A]	Attractor Experiment	Multi-perceptons
If_{nmda}	8.9e-9	8.9e-9
If_{dc}	0e-12	0e-12
If_{thr}	35e-12	5.2e-12
If_{tau}	30e-12	10e-12
If_{casc}	0e-12	0e-12
If_{ahtau}	4.5e-9	4.5e-9
If_{ahw}	0e-12	0e-12
If_{rfr}	3e-9	3e-9

Table 2: Soma-learning bias currents

Current value [A]	Attractor Experiment	Multi-perceptons
sl_{cathr}	80e-11	80e-11
sl_{catau}	6e-12	6.5e-12
sl_{caw}	8e-6	24e-6
sl_{wta}	0.2e-6	0.2e-6
sl_{thmin}	0.02n	2e-8
sl_{thup}	0.2e-6	2.8e-6
sl_{thdn}	0.2e-6	1.1e-6

Table 3: Short-term bias currents

Current value [A]	Attractor Experiment	Multi-perceptons
wht_{exc}	14e-9	2.5e-6
wht_{exc0}	0.19e-6	0.2e-7
wht_{exc1}	0.4e-6	10e-7
wht_{inh}	1.25e-6	20.5e-7
wht_{inh0}	18.8e-6	40e-7
wht_{inh1}	1.9e-9	3.4e-8

Table 4: Long-term plasticity synapse bias currents

Current value [A]	Attractor Experiment	Multi-perceptons
$pa_{deltaup}$	0e-12	20e-9
$pa_{deltadn}$	0e-12	20e-9
pa_{tail}	105e-9	8.6e-6
$pa_{driftup}$	31.5e-12	8.6e-12
$pa_{driftdn}$	40e-12	8.5e-12
pa_{wht}	23e-6	19.2e-6
pa_{whtr}	1.9e-9	8.6-6

REFERENCES

J. Sparsø (2001), Asynchronous circuit design - A tutorial, Chapters 1-8 in Principles of asynchronous circuit design - A systems Perspective, *Kluwer Academic Publishers*, 1–152
